# Melanoma-associated fibroblasts impair CD8+ T cell function and modify expression of immune checkpoint regulators via increased arginase activity

**DOI:** 10.1007/s00018-020-03517-8

**Published:** 2020-04-23

**Authors:** Barbara Érsek, Pálma Silló, Ugur Cakir, Viktor Molnár, András Bencsik, Balázs Mayer, Eva Mezey, Sarolta Kárpáti, Zoltán Pós, Krisztián Németh

**Affiliations:** 1 Department of Genetics, Cell and Immunobiology, Semmelweis University, 4 Nagyvarad ter, VII/709, Budapest, 1089 Hungary; 2 Office for Research Groups Attached to Universities and Other Institutions of the Hungarian Academy of Sciences, Budapest, 1051 Hungary; 3 Department of Dermatology, Venereology and Dermatooncology, Semmelweis University, Budapest, 1085 Hungary; 4 Institute of Genomic Medicine and Rare Disorders, Semmelweis University, Budapest, 1083 Hungary; 5 National Institute of Dental and Craniofacial Research, National Institutes of Health, Bethesda, MD 20815 USA

**Keywords:** Melanoma, Cancer-associated fibroblasts, Immunosuppression, T lymphocytes, cytotoxic, Arginase, Tumor microenvironment

## Abstract

**Abstract:**

This study shows that melanoma-associated fibroblasts (MAFs) suppress cytotoxic T lymphocyte (CTL) activity and reveals a pivotal role played by arginase in this phenomenon. MAFs and normal dermal fibroblasts (DFs) were isolated from surgically resected melanomas and identified as Melan-A-/gp100-/FAP+ cells. CTLs of healthy blood donors were activated in the presence of MAF- and DF-conditioned media (CM). Markers of successful CTL activation, cytotoxic degranulation, killing activity and immune checkpoint regulation were evaluated by flow cytometry, ELISPOT, and redirected killing assays. Soluble mediators responsible for MAF-mediated effects were identified by ELISA, flow cytometry, inhibitor assays, and knock-in experiments. In the presence of MAF-CM, activated/non-naïve CTLs displayed dysregulated ERK1/2 and NF-κB signaling, impeded CD69 and granzyme B production, impaired killing activity, and upregulated expression of the negative immune checkpoint receptors TIGIT and BTLA. Compared to DFs, MAFs displayed increased amounts of VISTA and HVEM, a known ligand of BTLA on T cells, increased l-arginase activity and CXCL12 release. Transgenic arginase over-expression further increased, while selective arginase inhibition neutralized MAF-induced TIGIT and BTLA expression on CTLs. Our data indicate that MAF interfere with intracellular CTL signaling via soluble mediators leading to CTL anergy and modify immune checkpoint receptor availability via l-arginine depletion.

**Graphic abstract:**

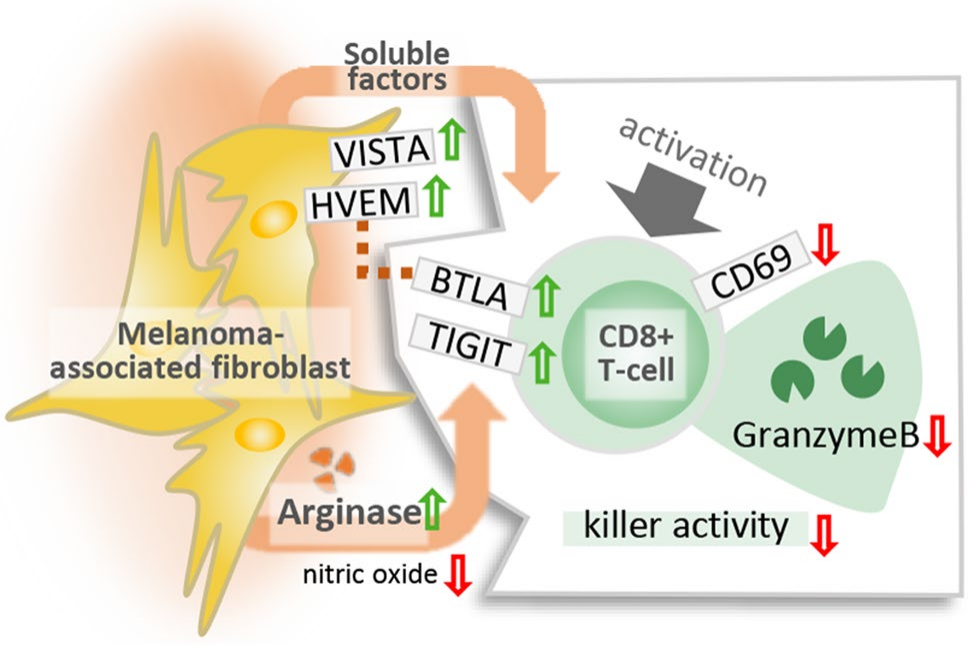

**Electronic supplementary material:**

The online version of this article (10.1007/s00018-020-03517-8) contains supplementary material, which is available to authorized users.

## Introduction

Cancer-associated fibroblasts (CAF) represent a heterogeneous cell population, considered to originate from various possible precursors, such as resting resident fibroblasts, bone marrow-derived mesenchymal stem cells, hematopoietic stem cells, endothelial cells, local epithelial cells, or adipocytes [1]. CAFs contribute actively to tumor progression by promoting matrix remodeling, invasion and neovascularization. CAFs are also known to suppress local immune responses via a wide array of cytokines, small molecular mediators and metabolic enzymes, such as transforming growth factor beta (TGFβ), IL-6, prostaglandin E2 (PGE2), VEGF and indoleamine-2,3-dioxygenase (IDO)/kynurenine [2, 3]. The relevance of CAFs in local immunosuppression is underlined by multiple observations showing that their removal may prolong survival or achieve immune-mediated rejection of established tumors [4–7].

CAFs can be found in virtually all solid tumors; in some, they are highly abundant (e.g., ovarian cancer); while in others, they are less frequent (e.g., melanoma). In melanoma, it is well documented that CAF (melanoma-associated fibroblasts, MAF) are rather capable suppressors of NK-cell activity via multiple ways, including PGE2-dependent suppression [8–10], and this phenomenon is probably not restricted to melanoma [11, 12].

Interestingly, it has been shown that activated effector CD8+ T cells express the same prostaglandin receptors that were reported to be involved in MAF-mediated NK-cell suppression. Also, CD8+ T cells exhibit decreased interferon gamma (IFNγ), tumor necrosis factor alpha (TNFα) and IL-2 production and impaired survival upon exposure to PGE2 [13]. However, it has not yet been analyzed whether MAF could influence CD8+ CTL responses via PGE2, via the release of immunosuppressive cytokines, metabolic reprogramming, immune checkpoint modulation, or other ways of immunosuppression. Considering that (i) CD8+ T cells play a pivotal role in melanoma rejection, (ii) state-of-the art melanoma therapies may achieve partial to complete patient responses by mobilizing CTLs, and (iii) factors contributing to therapy failure remain elusive, in this study, we sought to analyze possible parallels between MAF action on NK cells and CTLs, focusing on the latter.

## Materials and methods

### Patients and samples

Skin biopsies of thirteen melanoma patients were collected for research purposes following IRB (Institutional Review Board)-approved protocols, after obtaining informed consent, at the Department of Dermatology, Venereology and Dermatooncology, Semmelweis University, Budapest, Hungary. The study was conducted according to the Declaration of Helsinki principles and approved by the Hungarian Scientific and Research Ethics Committee of the Medical Research Council (ETT TUKEB; Decree No. 32/2007, supplements 32-2/2007 and 32-3/2007).

### Fibroblasts

Melanoma-associated fibroblasts (MAFs) were isolated from either primary or metastatic tumors of melanoma patients. The inner tumor mass was minced into ~ 1 mm^3^ pieces and digested in 20 ml DMEM supplemented with 200 U/ml type IV collagenase and 0.6 U/ml dispase (Thermo Scientific, 17104019, 17105041) as described elsewhere [14, 15]. MAFs were separated from melanoma cells by differential adhesion and their purity was assessed by flow cytometry. Normal dermal fibroblasts (DFs), serving as controls, were derived from non-tumorous intact edges (tissue > 1 cm from the tumor edge) using the same approach. Isolation of both MAFs and DFs from the same patient was successful in nine cases. These autologous, matched pairs of primary cells (see Supplementary table 1) were used in all subsequent analyses. Cells were used at five or fewer passages and maintained in standard DMEM (Gibco, 31885-023) supplemented with 20% FBS (Gibco, 10437-036), 1% penicillin–streptomycin (P/S) and 1% l-glutamine (Sigma-Aldrich, P4333, 59202C).

### Preparation of DF- and MAF-conditioned media

Viable isolated DF and MAF cultures reaching 75–80% confluence were washed twice in 1 × PBS, and further cultivated in 10-ml basal medium (BM) consisting of DMEM, 1% P/S, 1% l-glutamine and 0.5% BSA (Miltenyi Biotec, 130-091-376) for 48 h to prepare conditioned media (CM). In some experiments, BM were supplemented with 500-μM BEC (*S-*(2-boronoethyl)-l-cysteine) hydrochloride (Sigma-Aldrich, SML1384) to suppress arginase 1 and 2 activity. Finally, conditioned medium samples were centrifuged at 4000×*g* for 10 min, filtered through a 0.45-μm strainer, aliquoted and stored at − 80 °C until further use.

### MAF transfection

One million MAF cells were transfected with 1 ug of pEZ-M73 plasmid DNA containing the full-length human arginase-2 ORF under the control of a CMV promoter (Tebu-Bio EX-M0204-M73). Transfection was done by electroporation using a P2 Primary Cell 4D-Nucleofector™ X Kit (Lonza V4XP-2024) and a 4D Nucleofector (Lonza). Successful transfection was confirmed by Q-PCR. Conditioned media were prepared and applied as above.

### Cultivation of CD8+ T cells in DF- and MAF-conditioned media

Whole blood samples were collected from healthy volunteers (see Supplementary Table 2) by venipuncture in ACD A tubes (Greiner Bio-One, 455055). PBMC were isolated over Ficoll–Histopaque (Sigma, 10771). Separation of untouched human CD8+ T cells was carried out by negative magnetic selection using the Human CD8+ T Cell Isolation Kit (Miltenyi, 130-096-495) and an AutoMACS Pro II automated cell sorter (Miltenyi). T cells were counted and seeded on 48-well non-treated culture plates at a density of 1 × 10^6^ cells/well, in a 1:1 mixture of 250 ul of MAF- or DF-derived CM, and 250 ul X-vivo 15 medium (Lonza, 04-418Q) supplemented with 1% P/S, glutamine and 5% normal human male AB serum (Sigma-Aldrich, H4522).

### Activation of CD8+ T cells in DF- and MAF-conditioned media for downstream analyses

Unless otherwise stated, T cells were incubated in CM for 24 h before activation. Subsequently, T cells were distributed into 96-well round-bottom plates that were previously coated with 5 ug/ml anti-human CD3 (UCHT1; BD Biosciences, 555329) without changing the culture media. Next, 5 ug/ml anti-human CD28 (CD28.2; BD Biosciences, 555728) was added and cells were maintained for 48 h before downstream analyses were performed.

### Flow cytometry

Cells were stained with primary and secondary antibodies in pre-determined dilutions at 4 °C for 15 min (see Supplementary Table 3). Intracellular antigens were stained after cell surface antigens, using Sony’s Fixation and Intracellular Staining Permeabilization Wash Buffers for cytoplasmic, (Sony, 2704005, 2705010), and eBioscience’s Foxp3/Transcription Factor Staining Buffer Set for nuclear proteins (Thermo, 00-5523-00). Cytokines and secretory molecules were analyzed after Brefeldin A treatment (eBioscience, 00-4506-51); 5 µg/ml for 2 h at 37 °C before staining. Intracellular nitric oxide (NO) levels were determined using DAF-FM staining (Thermo Fisher, D23844), as recommended by the manufacturer.

### Signaling assays

MACS-sorted CD8+ T cells were cultivated in the presence of DF- and MAF-CM as above. Subsequently, T cells were exposed to a-CD3 (HIT3a) and a-CD28 (CD28.2) for 30 min, on wet ice, in the original cell culture media, and then washed twice with ice-cold PBS and finally activated by adding AffiniPure F(abʹ)2 Fragment Goat Anti-Mouse IgG, F(ab')2 fragment-specific crosslinker antibody (Jackson ImmunoResearch, 115–006-006) in DMEM at 37 °C for 10 min. Cells were washed with ice-cold 1 × PBS, fixed and stained for flow cytometry, as above. In other experiments, cells were lysed and analyzed by a Proteome Profiler Human Phospho-Kinase Array Kit (R&D, ARY003B), as per the manufacturer’s instructions. Specific signals were detected by a ChemiDoc XRS + system (Bio-Rad), and normalized to Hsp60 as internal reference.

### Redirected killing assay

Redirected killing assays were performed on MACS-sorted CD8+ T cells exposed to DF-, or MAF-conditioned media for 24 h in the presence or absence of anti-CD3/28 [16, 17]. P815 mouse mastocytoma cells, used as targets, were maintained in standard DMEM, 10% FBS, 1% P/S and l-glutamine. For redirected killing assays, equal amounts of P815 cells (1 × 10^6^/ml/assay) were stained with DiD (1,1′-dioctadecyl-3,3,3′,3′-tetramethylindodicarbocyanine, 4-chlorobenzenesulfonate salt) and DiL (1,1′-dioctadecyl-3,3,3′,3′-tetramethylindocarbocyanine perchlorate) dyes, respectively (Thermo Fisher, V22887, V22885). After extensive washing, DiD-stained cells, serving as specific targets, were loaded with mouse anti-human CD3 (eBioscience, 555336) and mouse anti-human CD28 (BD Biosciences, 555728) antibodies capable of activating CD8+ T cells. DiL-stained cells, serving as non-specific target controls were left unloaded. Target and non-target P815 cells were mixed at a ratio of 1:1 and co-cultured with CD8+ T cells at a ratio of 10:1 on 96-well U-bottom plates for 5 h. Subsequently, cultures were stained with Annexin V FITC (Biotium, 29005), and specific and unspecific killing was assessed by flow cytometry, based on annexin V staining of DiD- and DiL-labeled P815 cells, respectively. Percent specific lysis was calculated, as follows: 100 × (lysis of specific targets − lysis of non-specific target controls)/(100 – lysis of non-specific target controls).

### ELISA

Levels of TGFβ-1, IL-6, Prostaglandin E2, l-kynurenine, and SDF-1/CXCL12 in MAF and DF supernatants were comparatively analyzed by dedicated ELISA assays following the manufacturers’ instructions (see detailed assay information in Supplementary Table 3).

### ELISPOT

IFN-γ and granzyme B release was measured with the Human IFN-γ/Granzyme B Dual-Color ELISpot Kit (see detailed assay information in Supplementary Table 3). CD8+ T cells were isolated as described previously, and incubated in MAF- or DF-derived CM for 24 h. Next day, 1 × 10^5^ primed T cells were placed in each well of the ELISPOT plate in duplicates. Cells were activated with 5 ug/ml anti-human CD3 (HIT3a) and anti-human CD28 (CD28.2) for 48 h, or left untreated. Results were evaluated according to the manufacturer’s instructions, using an Alpha CTL Immunoscan ELISPOT reader.

### Q-PCR

Total RNA was isolated from fibroblasts using miRNeasy Kit (Qiagen 217004). Five hundred-ng RNA was reverse transcribed with a High-Capacity RNA-to-cDNA Kit (Thermo 4387406). A human Arginase-2 Taqman probe set (Thermo 4331182) was used to perform RT-Q-PCR on a 7900HT Sequence Detection System (Thermo).

### MAF arginase activity assay

Evaluation of arginase activity was carried out using a colorimetric Arginase Activity Assay Kit (Sigma-Aldrich, MAK112) following the manufacturer’s instructions. Results were evaluated according to the manufacturer’s instructions and normalized to total protein content.

### Statistical analysis

All statistical analyses were carried out with Graphpad Prism v7.00. Normality and equal variance were checked using Shapiro–Wilk tests and *F* tests, respectively. Normally distributed data with equal variance were analyzed using paired Student’s *t* tests, ratio paired *T* tests, unpaired *t* tests, one-way or two-way repeated measures ANOVA. All other datasets were compared using Mann–Whitney rank-sum tests, Wilcoxon matched-pairs rank-sum tests, or Friedman’s test, as appropriate. Differences were considered significant if *p* < 0.05. Sample sizes (*n*) indicated in figure legends refer to the actual number of independent melanoma patients (MAF, DF) and blood donors (CD8+ T cells) recruited in the experiment. Unless otherwise indicated (see Fig. 7), no MAF cultures, DF cultures, conditioned media or CD8+ T cell isolates were re-used in multiple experiments summarized in the same figure panel. In paired experimental designs and repeated measures studies, the same fibroblast culture or T cell isolate received multiple different treatments, or their combinations.

## Results

### Isolation and characterization of MAFs

**Fig. 1:**
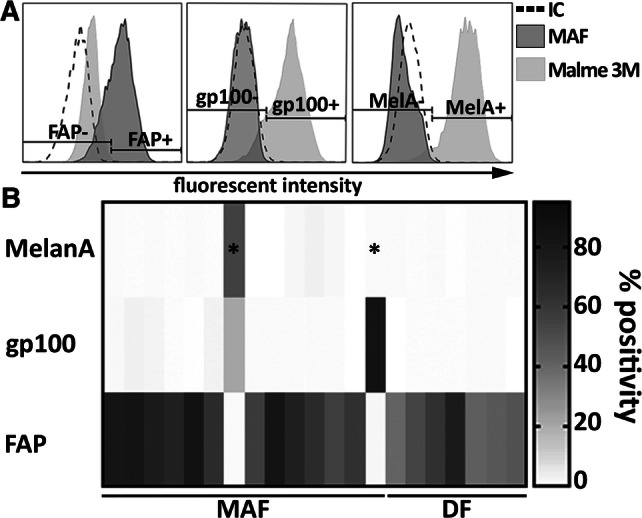
Flow cytometric characterization of primary fibroblast cultures obtained from resected melanoma samples. **a** Representative flow cytometry plots showing typical staining of validated, melanoma-free MAF cultures and Malme 3M melanoma cells used as reference. Specific staining with FAP-, GP100- and Melan-A antibodies is shown compared to isotype control (IC). **b** A heat plot summarizes expression patterns of all three markers, as observed on isolated MAF (*n* = 19) and DF samples (*n* = 11). Two primary MAF cultures out of fifteen, indicated by stars, were identified as melanoma-contaminated and censored. Summarized data from six independent measurements

Fibroblasts were isolated from cutaneous melanomas (MAFs) or from the healthy distal edges of the same surgical specimen, following elliptical excision (DFs, see Supplementary Table 1). Next, primary cultures of *bona fide* fibroblasts were extensively characterized by flow cytometry to validate their identity and exclude samples contaminated by melanoma cells. A three-marker panel was used, targeting melanoma-specific (melanA and gp100) and CAF markers (fibroblast-activation protein or FAP) (Fig. 1). MAF cultures displaying > 3–4% melanA or gp100 positivity were excluded from further experiments (Fig. 1, marked with asterisks); two such cultures were found out of sixteen.

### Early events of CD8+ T cell activation are compromised by MAF supernatants

**Fig. 2:**
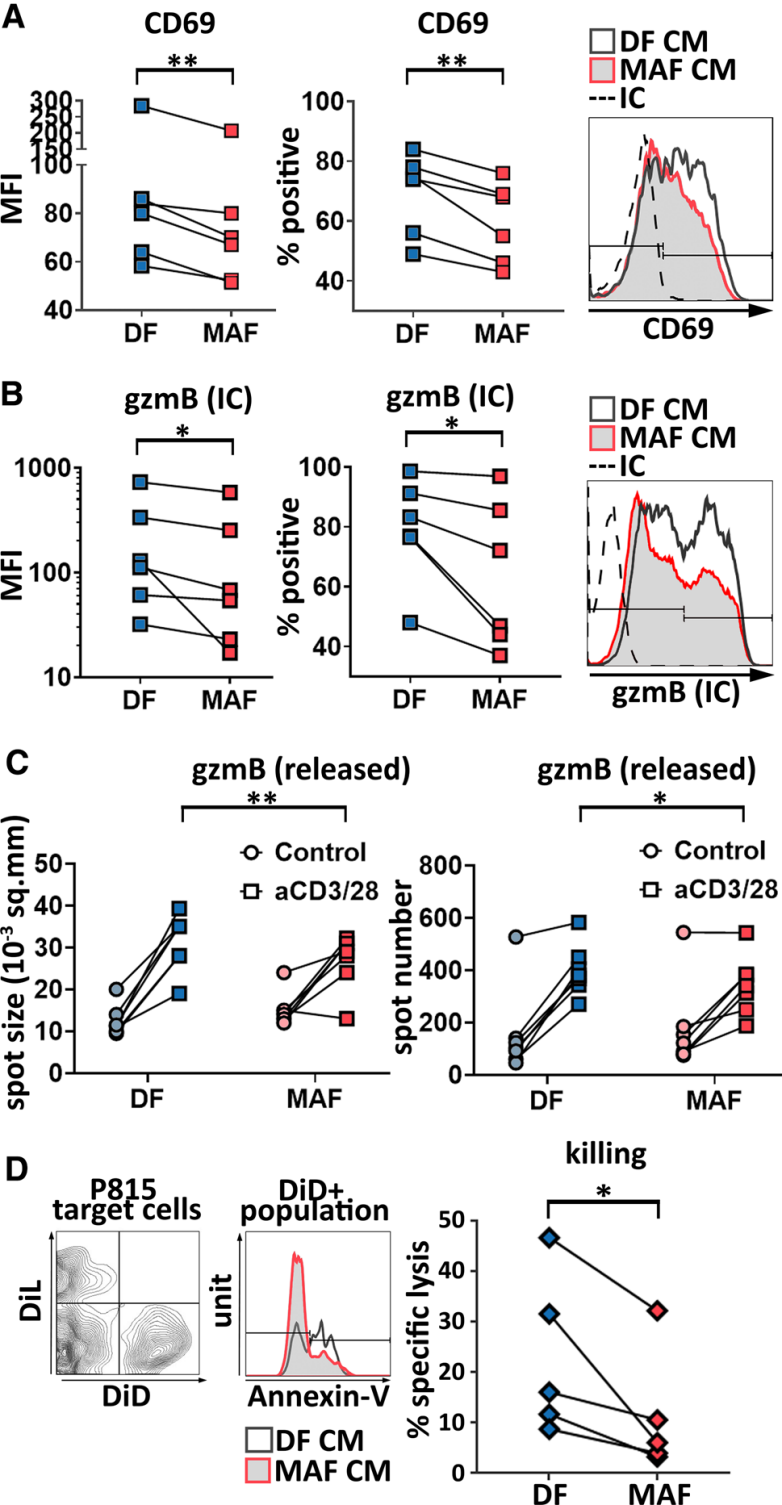
MAF-CM interferes with CD8+ T cell activation, intracellular granzyme B production, granzyme B release, and ex vivo killing. **a** Expression of CD69 was examined upon exposition of healthy blood donors’ CD8+ T cells to MAF- or DF-derived conditioned media and their subsequent activation by anti-CD3/CD28. Comparative analysis of mean fluorescence intensities (MFI) by ratio paired *t* test (*p* = 0.0053; *n* = 6), and percentage of CD69 positive cells analyzed by paired *t* test (*p* = 0.0058; *n* = 6) (result of two independent experiments) and representative staining. **b** Representative flow cytometry data displaying intracellular (IC) gzmB staining of split CD8+ T cell cultures activated by aCD3/28 after exposition to MAF-CM or DF-CM. Pairwise analysis of mean fluorescence intensity and percent positive cells are shown (paired *t* test *p* = 0.0346, *p* = 0.0327, *n* = 6 respectively; three independent experiments). **c** Comparative analysis of gzmB release (spot area) and gzmB-releasing cell numbers (spot number) in control and aCD3/28-treated CTLs after exposition to MAF-CM and DF-CM using ELISPOT assays (two-way RM ANOVA, *p* = 0.0409, *p* = 0.0050, respectively, *n* = 7, one experiment). **d** Redirected killing assay using P815 cells pre-stained with DID and pre-loaded with aCD3/28 (specific lysis targets) and their DIL-stained but otherwise untreated counterparts (non-specific lysis controls). Lysed P815 cells were identified using annexin V-staining and data were analyzed by ratio paired *t* test (*p* = 0.0223, *n* = 5) (two independent experiments). Representative staining and data evaluation are shown

We first tested whether early steps of CD8+ T cell activation were affected by the presence of MAF-released soluble factors. To this end, we examined whether MAF-CM would affect expression of either CD69, an early T cell activation marker, or that of CD107a (LAMP-1), a marker of cytotoxic degranulation. We found that CD8+ T cells activated by anti-CD3/28 in the presence of MAF-CM displayed less CD69 compared to CD8+ T cells activated in the presence of DF-CM, as the frequency of CD69+ cells was decreased. Similarly, the ratio of CD69(+) vs. CD69(−) cells decreased significantly in the MAF-CM vs DF-CM group (Fig. 2a). No significant difference was found in the amounts of cell surface CD107a, however (not shown).

### Selective impairment of CD8+ T cell effector molecule production and release by MAF supernatants

Next, we investigated if MAF-derived soluble factors could modulate expression of cytotoxic T cell effector molecules or killing activity. We tested the activation-induced production of IFN-γ and granzyme B (gzmB) by intracellular flow cytometry. There was no significant difference between MAF- and DF-instructed CD8+ T cells in terms of IFN-γ production (not shown). However, intracellular gzmB levels were significantly reduced in the presence of the MAF CM in CD8+ T cells compared to DF CM-treated cells. Similar to CD69, both gzmB production and the frequency of gzmB-positive cells (Fig. 2b) were reduced. Impact of MAF-CM on CTLs’ gzmB production was comparable to that of in vitro exposition to TGF-β in the dose range studied, and this held true regardless of the presence or absence of fibroblast-conditioned media (see Supplementary Fig. 1). Subsequent analysis of the release of gzmB and IFN-γ from MAF-exposed CTLs by ELISPOT corroborated these findings. ELISPOT confirmed that both the number of aCD3/28-activated gzmB-secreting CD8+ T cells, and the amount of gzmB released by them were reduced by pre-incubaton with MAF-CM (Fig. 2c). However, in line with flow data, IFN-γ release was not affected by MAFs (not shown).

### MAF-derived soluble factors hamper CD8+ T cell-mediated killing

As gzmB production and release correlate with cytotoxic T cell killing capacity, in vitro cytotoxic effector functions of activated CD8+ T cells were also analyzed in the presence of MAF-CM. Standard redirected killing assays [18–22] were performed using P815 cells serving as targets, either preloaded with anti-CD3/28 antibodies and labeled with DiD (targets to assess specific killing) or left untreated and labeled with DiL (to assess aspecific killing of bystander non-target cells). We observed that the MAF CM-treated CD8+ T cell group induced significantly less cell death than the DF CM-treated group (Fig. 2d), which is in line with the observed attenuation of gzmB production.

### MAFs interfere with T cell signaling

**Fig. 3:**
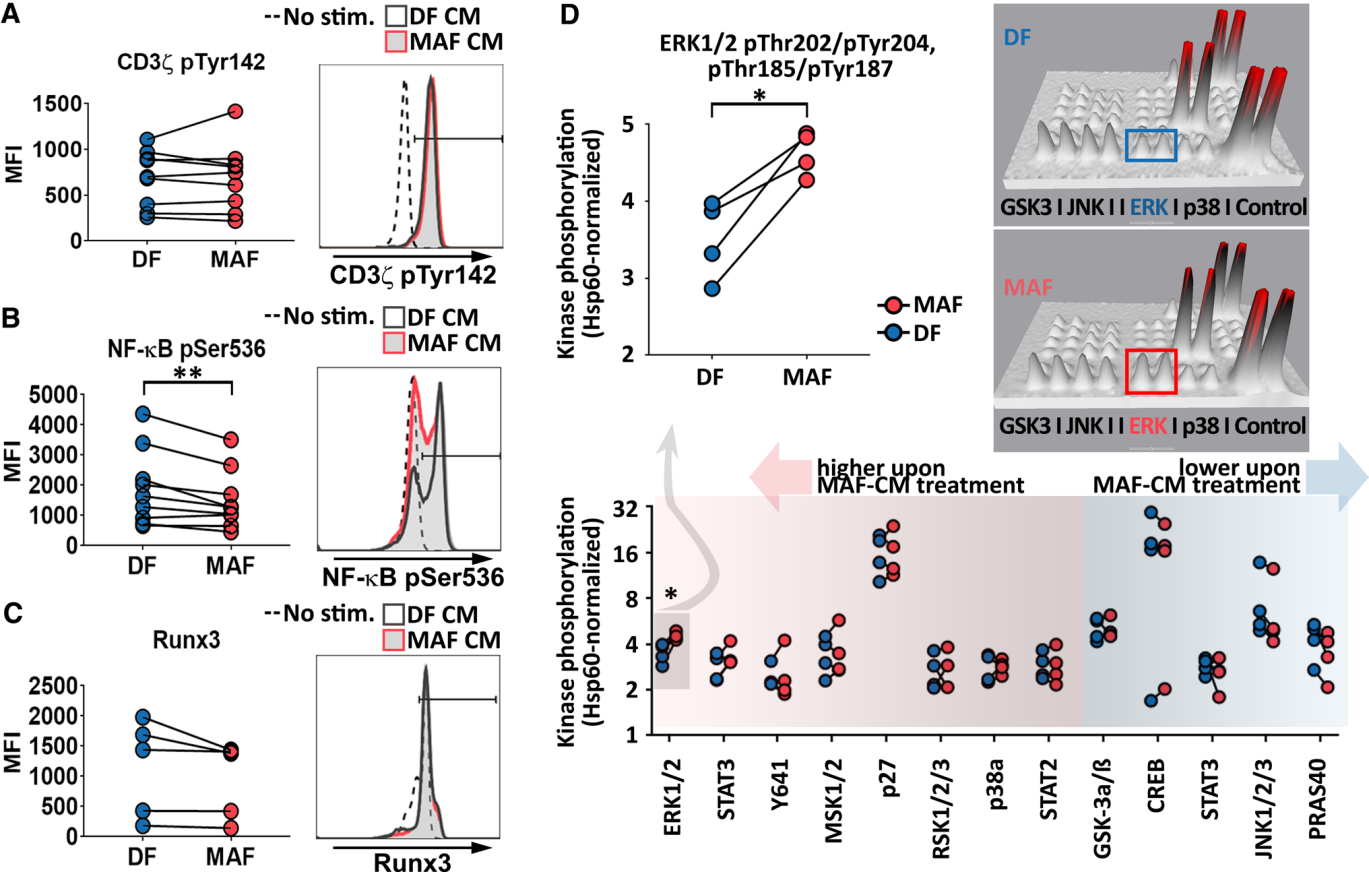
MAF-CM modulates CTL phosphorylation cascades downstream of TCR. **a** Analysis of CD3ζ-chain activation (*n* = 9). **b** NF-κB activation (*n* = 9) and **c** Runx3 levels (*n* = 5) in unstimulated, and DF-CM- or MAF-CM-exposed CD8+ T cells upon activation by aCD3/28. CD3ζ-chain and NF-κB phosphorylation were assessed 10 min post activation; Runx3 levels 48 h post activation. Representative data are shown to the right (Ratio paired *t* test *p* = 0.0093 for NF-κB; results from two independent experiments). **d** Screening analysis of 43 additional phosphorylated kinases in DF- and MAF-CM-treated CD8+ T cells using a phosphokinase array (*n* = 4). Shown are phosphorylation patterns of thirteen typical CD8+ T cell signaling proteins detected by the array 10 min post T cell activation (bottom). ERK1/2 phosphorylation data are shown magnified (top left, paired *t* test *p* = 0.0147). Representative 3D densitometry data display differences in ERK1/2 activation are shown (top right). Each phosphorylated protein is represented by a pair of spikes; volume of spikes equals to intensity of protein phosphorylation. Results obtained from one experiment

Considering that impaired T cell function may be caused by lack of proper co-stimulation or other forms of aberrant signal transduction, we next analyzed if MAF supernatants influenced T cell signaling, required for full T cell activation and differentiation. In pilot studies, we interrogated early (CD3ζ-chain activation, Tyr142-P), and downstream of T cell signaling events (NF-κB activation, Ser536-P) as well as a key transcription factor associated with late CD8+ T cell differentiation (Runt-related transcription factor 3 or Runx3). MAFs suppressed NF-κB phosphorylation, while CD3ζ and Runx3 remained unaffected (Fig. 3a–c). Based on this observation, we next utilized phospho-kinase protein arrays to gain further insight into aberrant T cell signaling downstream of TCR. Although the majority of TCR-related kinase pathways remained functional, suppression of NF-κB activation was accompanied by increased ERK1/2 phosphorylation in MAF-exposed CD8+ T cells (Fig. 3D). Taken together, these data suggest that there is an imbalance between aCD3-mediated TCR signaling and aCD28-provided co-stimulation in MAF CM-exposed T cells.

### MAF supernatants modify immune checkpoint receptor expression of CD8+ T cells

**Fig. 4:**
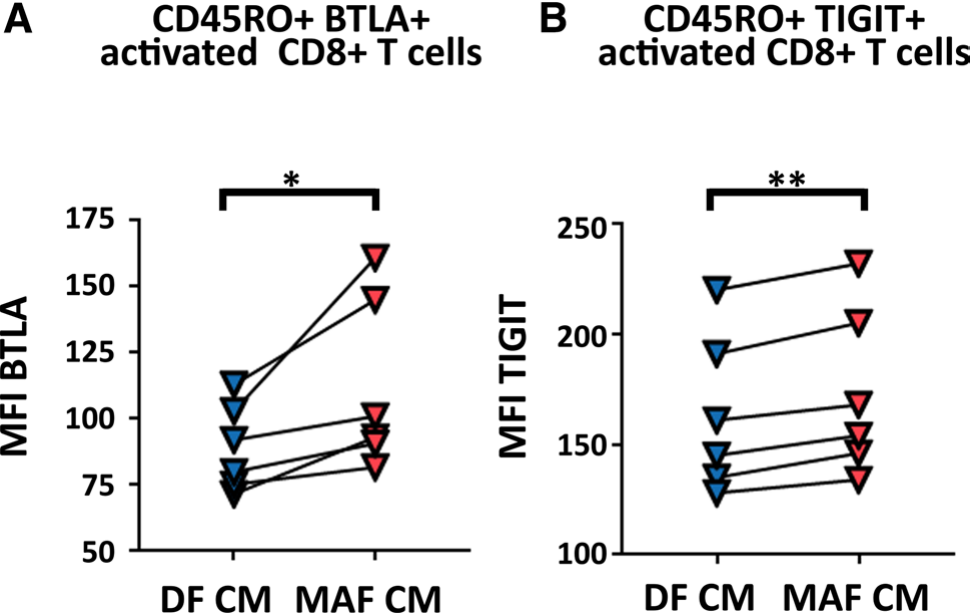
MAF-CM-treated activated non-naïve CD8+ T cells increase BTLA and TIGIT checkpoint receptor expression. BTLA and TIGIT expression on activated non-naïve/memory CD45RO+ CD8+ T cells upon exposition to DF- and MAF-conditioned media; paired *t* test (BTLA: *p* = 0.0353 and TIGIT: *p* = 0.0050, *n* = 6, data from one experiment

Immune checkpoint receptors (ICR) are known to regulate CD8+ T cell activity during anti-tumor immune responses. Therefore, we wanted to explore if MAFs can influence the synthesis of select ICRs in CD8+ T cells. Expression patterns of PD-1 (programmed cell death protein 1), TIM-3 (T cell immunoglobulin and mucin-domain containing-3), LAG-3 (lymphocyte-activation gene 3), TIGIT (T cell immunoreceptor with Ig and ITIM domains) and BTLA (B and T lymphocyte attenuator) were analyzed after treatment with MAF and DF-CMs. We found an increased expression of TIGIT and BTLA in CD45RO+ non-naïve/memory cytotoxic T cells following exposition to MAF-CM as compared to DF-CM (Fig. 4). The expression of PD-1, TIM-3, or LAG-3 on CD8+ T remained unaffected by MAF-CM compared to DF-CM, regardless of aCD3/28 activation or naïve/memory status (not shown).

### MAFs exhibit a skewed spectrum of immune checkpoint regulators

**Fig. 5:**
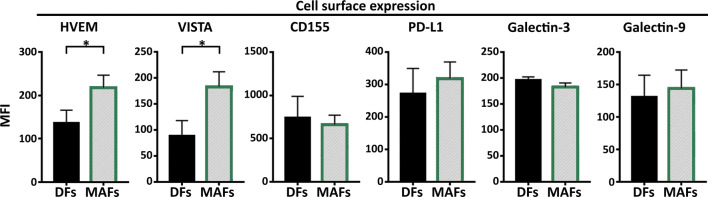
MAF maintain increased expression of the immune checkpoint regulators HVEM and VISTA. Comparative analysis of cell surface Herpesvirus entry mediator (HVEM), Galectin-3, Galectin-9, Programmed death-ligand 1 (PD-L1), CD155 (poliovirus receptor) and V-domain Ig suppressor of T cell activation (VISTA) expression (mean fluorescence intensity) on MAF and DF cells, as analyzed by flow cytometry (*n* = 6 Mann–Whitney *U* test BTLA *p* = 0.029 and HVEM *p* = 0.0311). Data from three independent experiments

The expression of CAF-ICR ligands and the possible role of these molecules in CAF-mediated immunomodulation have been controversial. To analyze the question whether MAF could affect T cell ICR signaling, cell surface expression of several ICR ligands was compared between MAFs and DFs. We tested the presence and expression of Herpesvirus entry mediator (HVEM), Galectin-3, Galectin-9, Programmed death-ligand 1 (PD-L1), CD155 (poliovirus receptor) and V-domain Ig suppressor of T cell activation (VISTA). We found that compared to DFs, MAFs displayed increased amounts of the negative CTL regulators VISTA and HVEM (Fig. 5).

### MAFs display increased l-arginase activity and secrete elevated levels of CXCL12

**Fig. 6:**
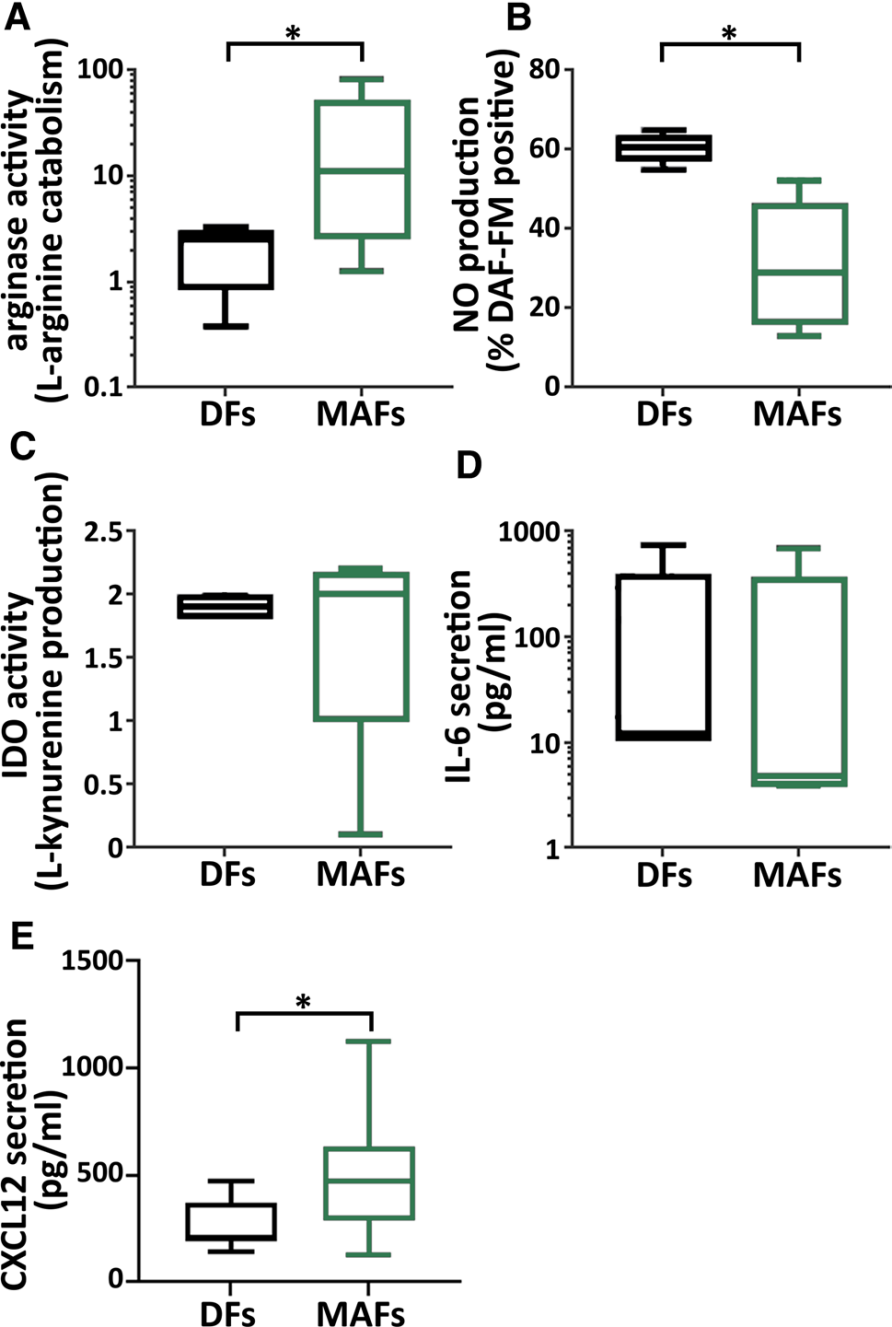
Increased arginase activity and apparent NOS uncoupling in MAF cells. **a** Intracellular arginase activity of MAF and DF cells derived from the same patients were compared in cell lysates. Results shown were normalized to total protein content (min to max; *p* = 0.0186, *n* = 5, ratio paired *t* test) (data from two independent experiments). **b** MAF and DF cells from the same patients were stained with DAF-FM and the frequency of NO producing cells was assessed (min–max; *p* = 0.0113, *n* = 5, paired *t* test, data from two independent experiments). Significant difference was found in the amount of produced NO (MFI) per cell, too (*p* = 0.0021, data not shown). **c**, **d** Evaluation of IL-IDO activity/l-kynurenine production, IL-6 and CXCL12 release (paired *t* test, *p* = 0.0178, *n* = 8, one experiment) in MAF and DF cells by ELISA

We next sought to explore the molecular pathways involved in MAF CD8+ T cell interactions. Candidate molecules were selected based on previous reports and included immunomodulatory factors synthetized by CAFs. Secreted TGF-β [23], IL-6, CXCL12 [5] and PGE2 [8] were analyzed by ELISA in MAF-CM and DF-CM. l-arginine catabolism by intracellular l-arginase [24] was analyzed in cell lysates using a dedicated l-arginase activity assay. l-tryptophan catabolism by Indolamine-2,3-dioxygenase [25] was assessed by measuring kynurenine in the CM. l-arginase activity was detected in both MAF and DF and it was highly elevated in MAF compared to DF cells (**Fig. ****6****A)**. To corroborate this finding, we next analyzed NO production by intracellular DAF-FM staining in a similar manner, comparing MAF and DF cells. l-arginase and nitric oxide synthase (NOS) are in active competition for free l-arginine as their common substrate, and increased arginase activity was repeatedly shown to induce uncoupling of NOS resulting in decreased NO production [26]. Indeed, we found that MAF produced markedly less NO than DF (Fig. 6b), suggesting that l-arginase upregulation and consequent l-arginine depletion in MAF reach a critical level, sufficient to disrupt normal NO production in these cells. IL-6, and IDO activity were detected, but none of them showed any significant difference between MAFs and DFs (Fig. 6c, d). No detectable amounts of TGF-β and PGE2 were found in either MAF- or DF-CMs (not shown), which is consistent with our findings (Supplementary Fig. 1) that the presence or absence of MAF- or DF-CM did not modify the impact of external TGF-β on CTLs gzmB production. Interestingly, elevated levels of secreted CXCL12 were detected in MAF supernatants, which is in line with other reports that describe an elevated CXCL12 production by CAFs in both human and murine carcinomas [5, 27] (Fig. 6e).

### MAF-derived l-arginase induces increased expression of TIGIT and BTLA in CD8+ T cells

**Fig. 7:**
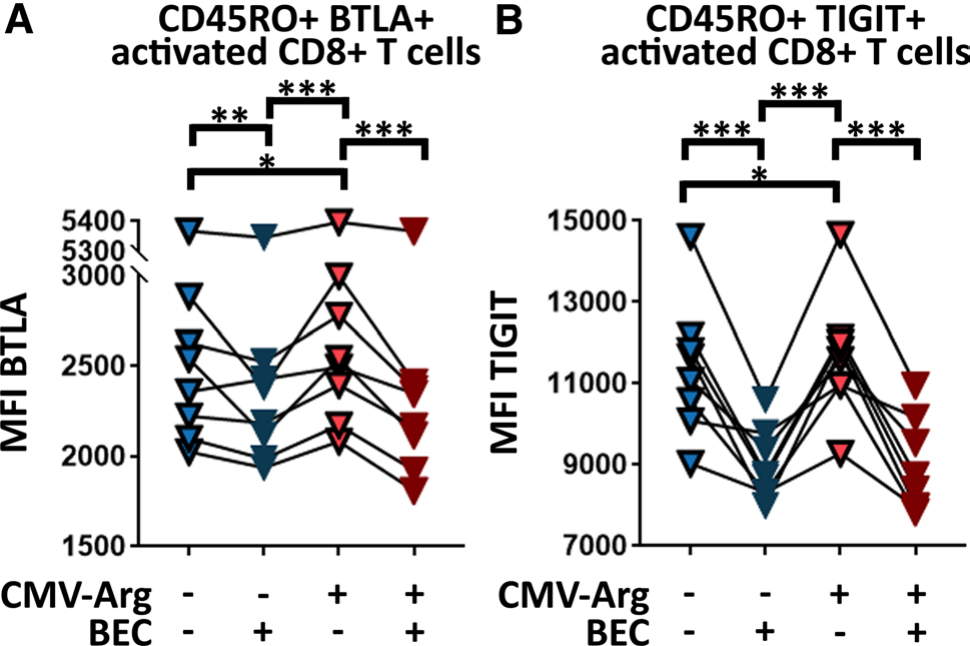
Increased MAF arginase activity is responsible for aberrant ICR expression on CTLs. Comparative analysis of ICR expression on activated non-naïve/memory CTLs exposed to conditioned media collected from MAF maintained in the presence and absence of BEC hydrochloride, a selective arginase inhibitor, and transgenic arginase overexpression (CMV-Arg). BTLA (**a**) and TIGIT (**b**) expression of activated CD45RO+ CD8+ T cells is shown under the influence of BEC and CMV-Arg. (two-way RM ANOVA, *p < 0.05, **p < 0.01 ***p < 0.001, *n* = 8). Data from two independent experiments

To test whether increased l-arginase activity in MAFs was responsible for MAF-induced CD8+ T cell suppression, additional batches of MAF-CM and DF-CM were prepared both in the presence and absence of BEC hydrochloride, a potent and selective inhibitor of both l-arginase 1 and 2. Next, all previously observed T cell-related functional aberrations were re-evaluated for stability with and without l-arginase inhibition. These analyses disclosed that MAF-suppressed CD69 and gzmB expression, and MAF-reduced CTL-mediated killing could not be restored by elimination of l-arginase activity in MAFs (Supplementary Fig. 2). However, the MAF-CM-elevated expression of BTLA and TIGIT could be neutralized by selective inhibition of l-arginase (Fig. 7a). To corroborate these findings, we transfected MAFs with a mammalian expression vector encoding the full-length human arginase 2 under the control of a CMV promoter (Supplementary Fig. 3), and examined its effect on both BTLA and TIGIT. We found that forced expression of arginase in MAFs further increased BTLA and TIGIT on CD8+ T cells (Fig. 7b). Taken together, these data indicate that MAF-mediated enhancement of BTLA and TIGIT expression on CD8+ T cells is an l-arginase-dependent phenomenon.

## Discussion

Research has shown that in the absence of T cell-activating immunotherapy, high counts of FAP + MAFs accumulate in the tumor stroma and this may serve as a negative prognostic marker [28]. This study provides multiple lines of evidence that soluble factors released by MAFs affect various aspects of cytotoxic T cell function. Our data suggest that ex vivo MAFs can create a milieu that influences virtually all phases of T cell activation ranging from early post-activation events to terminal differentiation and exertion of effector functions. Without exception, all observed effects are detrimental to CD8+ T cell activity. This raises the question whether MAFs in vivo were capable of contributing to the complex, multi-pronged attack mounted by cancer cells on CTLs, leading to the well-documented dysfunctionality of TILs in various advanced solid tumors, including melanoma.

Here, we show that FAP + melanoma-associated fibroblasts display an immunosuppressive, arguably tumor promoting phenotype, which is in line with observations of others made in colorectal [29] and pancreatic cancers [5]. MAF-derived soluble factors significantly downregulate the expression of CD69 on the surface of activated CD8+ T cells compared to healthy DFs, suggesting that fundamental perturbation of T cells occurs even at the earliest phases of T cell activation. MAF-derived factors reduce granzyme B production and release, but do not alter IFNγ production, secretion, or cytotoxic degranulation of CD8+ T cells, implying that selective impairment of distinct components of the T cell effector machinery may occur in the vicinity of MAFs. In line with this finding, in redirected killing assays, we demonstrate that MAFs suppress ex vivo killing capacity of CD8+ T cells too, which may be related to the attenuated granzyme B production. It is important to note, however, that experimental killing assays [16], including the redirected killing assays used here [17, 19, 30] measure ex vivo killing capacity, and shall be interpreted with caution, as their results may not necessarily reflect actual antitumor killing activity in vivo. Nevertheless, these findings suggest that MAFs may not only disturb the process of T cell activation, but also could undermine effective elimination of target cells (i.e., cancer cells) in the tumor parenchyma.

Moreover, we found selective dysregulation of distinct immune checkpoint regulators on CD8+ T cells by MAFs, as well. The ratios of TIGIT- and BTLA-positive CD8+ T cells were significantly elevated by soluble MAF factors, particularly among non-naïve/memory CD45RO+ T cells. This observation is intriguing because several ICRs, including BTLA [20] and TIGIT [31] are potent negative regulators of T cell activity and are actively researched as promising targets for next generation immunotherapies. Monoclonal antibodies targeting BTLA are subject of preclinical studies, while TIGIT blockade is in Phase I clinical trials (NCT03119428). Further, activity of CD45RO+ T cells is decisive in the rejection of various solid tumors [32–34], including checkpoint blockade therapy-induced remission of melanoma. Several lines of evidence indicate that frequency of circulating CD45RO+ T cells [35], and particularly therapy-induced systemic [36, 37] and intratumoral [38] expansion of the CD45RO+ subset are of major importance in the success of checkpoint blockade. Interestingly, changes observed in CD8+ T cell ICR expression (higher BTLA levels) were paralleled by complementary changes in MAF-derived ICR ligands (increased HVEM expression). The simultaneous up-regulation of HVEM and BTLA observed on MAFs and MAF-exposed CTLs, respectively, raises the question whether MAFs could further suppress CTLs by activating the HVEM-BTLA ICR signaling axis, should direct contact between the two cell types occur [39].

Related to the findings above, potential differences were searched between the MAF and DF cells that could shed light on the mechanisms responsible for the apparent dysfunction of MAF-exposed CD8+ T cells. We found that intracellular signaling cascades of CD8+ T cells were affected by prolonged exposition to soluble MAF mediators. Increased ERK1/2 and decreased NF-κB phosphorylation suggest that MAF reprogram two essential T cell signaling pathways, possibly the RAS guanyl-releasing protein 1–ERK1/2 pathway, and the protein kinase Cθ–NF-κB pathway. Mature naïve T cells activated by aCD3 (stimulation) and aCD28 (co-stimulation) rely on both pathways equally for TCR/CD3 signaling [40]. However, it has been extensively documented that sufficient CD28-induced co-stimulation depends on the protein kinase Cθ–NF-kB pathway [41-44]. As full T cell maturation and exertion of effector functions are both dependent on proper co-stimulation, these data indicate that the phenotypic changes observed in the effector functions of MAF-exposed CD8+ T cells may originate in weakened T cell co-stimulation.

Furthermore, we found that arginase activity was highly elevated in MAF cells, to an extent that it resulted in both uncoupling of NOS and clear disturbances of the steady-state NO production. l-arginine depletion induces T cell suppression by (i) down-regulation of CD3ζ [45], (ii) G0/G1 cell-cycle arrest due to failed upregulation of cyclin D3 protein synthesis and cdk4 activation [46], (iii) increased phosphorylation of eIF2 and (iv) global suppression of protein synthesis in affected T cells [47] (v) interference with lytic function in situ [48]. So far, direct evidence for CAFs inducing T cell suppression via arginase has not been provided, although clinical data clearly show that ARG2 overexpression by CAFs predisposes to poor overall survival in pancreatic cancer [24] and ARG2 production by CAF has been observed in unrelated solid tumors, too [49]. It has been proposed that cancer-associated fibroblasts may also exploit arginase to interfere with T cell activity [1, 2, 50]; however, to our best knowledge, this is the first report substantiating this hypothesis on the example of melanoma-associated fibroblasts.

We found that selective blockade of arginase activity by a potent, small molecular inhibitor, BEC hydrochloride, was able to neutralize the increased expression of TIGIT and BTLA on CD45RO+ CD8+ T cells 48 h after activation, while arginase transfection had the opposite effect. Collectively, these data suggest that aberrant ICR expression on CTLs in the presence of MAF-CM is an arginase-mediated phenomenon. Interestingly, a recent paper also suggests that BTLA may depend on arginase, as in the absence of arginase 2, BTLA mRNA expression decreases in activated CD8+ T cells 48 h upon activation [51]. Of note, BEC hydrochloride maintained its effectiveness even after MAF arginase expression was increased by stable transfection. This is interesting because arginase inhibitors, either alone or in combination with checkpoint inhibitors, are currently being tested as anti-neoplastic agents in various solid malignancies (NCT02903914). These clinical trials are in concert with preclinical data suggesting that arginase blockade may increase efficiency of the PD-1/PD-L1 blockade [52, 53]. Thus, our data may support the notion that arginase inhibition may be a tool that makes some solid tumors more vulnerable to multiple forms of ICR-blockade.

Last but not least, we demonstrated that MAFs secrete more CXCL12 as compared to autologous DF cells. High amounts of CXCL12 can act as a chemorepellent, and may be implicated in the exclusion of CD8+ T cells from solid tumors [5, 54]. Although multiple lines of evidence suggest that increased CXCL12 secretion by CAFs is pro-tumorogenic [27], further research is needed to explore to exact role of MAF-derived CXCL12 in the biology of melanoma.

One particularly intriguing aspect of our observations is the remarkable robustness of the CTL-suppressive MAF phenotype. In all experiments, MAFs were maintained in the absence of melanoma cells for several weeks before being used for CD8+ T cell assays. Our data clearly demonstrate that in spite of extensive in vitro cultivation, MAFs did not revert to the rather harmless DF phenotype. The CTL-suppressive MAF phenotype remained stable even after separation from the cancer microenvironment. This observation supports the notion put forward recently by many leading authors of the field, that at least some changes promoting the transition of DF to CAF may rely on highly stable epigenetic modifications [55–58]. That is, some parameters of the MAF phenotype may arise following a single priming event, resulting in long-term consequences, rather than being maintained by cancer cells via persistently secreted cytokines or similar mechanisms [1, 59]. Although the exact mechanism of this tumor-initiated transformation of fibroblasts is unknown, some studies suggest that direct cell–cell contact, as well as cell-derived soluble factors such as TGF-beta and LIF may play [55, 60] an important role in the this process.

## Electronic supplementary material

Below is the link to the electronic supplementary material.

Supplementary file1 (EPS 42796 kb)

Supplementary file2 (EPS 35155 kb)

Supplementary file3 (EPS 18161 kb)

Supplementary file4 (PDF 90 kb)

Supplementary file5 (PDF 119 kb)

Supplementary file6 (PDF 86 kb)

## Abbreviations

BEC: (*S*-(2-Boronoethyl)-l-cysteine) hydrochloride
BM: Basal medium
BTLA: B and T lymphocyte associated
CAF: Cancer-associated fibroblasts
CM: Conditioned media
CTL: Cytotoxic T lymphocyte
DF: Normal dermal fibroblasts
DiD: (1,1′-Dioctadecyl-3,3,3′,3′-tetramethylindodicarbocyanine, 4-chlorobenzenesulfonate Salt)
DiL: (1,1′-Dioctadecyl-3,3,3′,3′-tetramethylindocarbocyanine perchlorate)
FAP: Fibroblast-activation protein
GzmB: Granzyme B
HVEM: Herpesvirus entry mediator
ICR: Immune checkpoint receptors
IDO: Indoleamine-2,3-dioxygenase
IFNγ: Interferon gamma
LAG-3: Lymphocyte-activation gene 3
MAF: Melanoma-associated fibroblasts
NO: Nitric oxide
NOS: Nitric oxide synthase
PD-1: Programmed cell death protein 1
PD-L1: Programmed death-ligand 1
PGE2: Prostaglandin E2
Runx3: Runt-related transcription factor 3
TGFβ: Transforming growth factor beta
TIGIT: T cell Immunoreceptor with Ig and ITIM Domains
TIM-3: T cell immunoglobulin and mucin-domain containing-3
TNFα: Tumor necrosis factor alpha
VEGF: Vascular endothelial growth factor
VISTA: V-domain Ig suppressor of T cell activation

## References

[1] Kalluri R (2016). The biology and function of fibroblasts in cancer. Nat Rev Cancer.

[2] Ziani L, Chouaib S, Thiery J (2018). Alteration of the antitumor immune response by cancer-associated fibroblasts. Front Immunol.

[3] Shiga K, Hara M, Nagasaki T, Sato T, Takahashi H, Takeyama H (2015). Cancer-associated fibroblasts: their characteristics and their roles in tumor growth. Cancers (Basel).

[4] Kraman M, Bambrough PJ, Arnold JN, Roberts EW, Magiera L, Jones JO, Gopinathan A, Tuveson DA, Fearon DT (2010). Suppression of antitumor immunity by stromal cells expressing fibroblast activation protein-alpha. Science.

[5] Feig C, Jones JO, Kraman M, Wells RJ, Deonarine A, Chan DS, Connell CM, Roberts EW, Zhao Q, Caballero OL, Teichmann SA, Janowitz T, Jodrell DI, Tuveson DA, Fearon DT (2013). Targeting CXCL12 from FAP-expressing carcinoma-associated fibroblasts synergizes with anti-PD-L1 immunotherapy in pancreatic cancer. Proc Natl Acad Sci U S A.

[6] Wang LC, Lo A, Scholler J, Sun J, Majumdar RS, Kapoor V, Antzis M, Cotner CE, Johnson LA, Durham AC, Solomides CC, June CH, Pure E, Albelda SM (2014). Targeting fibroblast activation protein in tumor stroma with chimeric antigen receptor T cells can inhibit tumor growth and augment host immunity without severe toxicity. Cancer Immunol Res.

[7] Zhang Y, Ertl HC (2016). Depletion of FAP+ cells reduces immunosuppressive cells and improves metabolism and functions CD8+T cells within tumors. Oncotarget.

[8] Balsamo M, Scordamaglia F, Pietra G, Manzini C, Cantoni C, Boitano M, Queirolo P, Vermi W, Facchetti F, Moretta A, Moretta L, Mingari MC, Vitale M (2009). Melanoma-associated fibroblasts modulate NK cell phenotype and antitumor cytotoxicity. Proc Natl Acad Sci U S A.

[9] Pietra G, Manzini C, Rivara S, Vitale M, Cantoni C, Petretto A, Balsamo M, Conte R, Benelli R, Minghelli S, Solari N, Gualco M, Queirolo P, Moretta L, Mingari MC (2012). Melanoma cells inhibit natural killer cell function by modulating the expression of activating receptors and cytolytic activity. Cancer Res.

[10] Ziani L, Safta-Saadoun TB, Gourbeix J, Cavalcanti A, Robert C, Favre G, Chouaib S, Thiery J (2017). Melanoma-associated fibroblasts decrease tumor cell susceptibility to NK cell-mediated killing through matrix-metalloproteinases secretion. Oncotarget.

[11] Holt D, Ma X, Kundu N, Fulton A (2011). Prostaglandin E(2) (PGE (2)) suppresses natural killer cell function primarily through the PGE(2) receptor EP4. Cancer Immunol Immunother.

[12] Inoue T, Adachi K, Kawana K, Taguchi A, Nagamatsu T, Fujimoto A, Tomio K, Yamashita A, Eguchi S, Nishida H, Nakamura H, Sato M, Yoshida M, Arimoto T, Wada-Hiraike O, Oda K, Osuga Y, Fujii T (2016). Cancer-associated fibroblast suppresses killing activity of natural killer cells through downregulation of poliovirus receptor (PVR/CD155), a ligand of activating NK receptor. Int J Oncol.

[13] Chen JH, Perry CJ, Tsui YC, Staron MM, Parish IA, Dominguez CX, Rosenberg DW, Kaech SM (2015). Prostaglandin E2 and programmed cell death 1 signaling coordinately impair CTL function and survival during chronic viral infection. Nat Med.

[14] Furedi A, Toth S, Szebenyi K, Pape VF, Turk D, Kucsma N, Cervenak L, Tovari J, Szakacs G (2017). Identification and validation of compounds selectively killing resistant cancer: delineating cell line-specific effects from p-glycoprotein-induced toxicity. Mol Cancer Ther.

[15] Szebenyi K, Furedi A, Kolacsek O, Csohany R, Prokai A, Kis-Petik K, Szabo A, Bosze Z, Bender B, Tovari J, Enyedi A, Orban TI, Apati A, Sarkadi B (2015). Visualization of calcium dynamics in kidney proximal tubules. J Am Soc Nephrol.

[16] Zaritskaya L, Shurin MR, Sayers TJ, Malyguine AM (2010). New flow cytometric assays for monitoring cell-mediated cytotoxicity. Expert Rev Vaccines.

[17] Saverino D, Tenca C, Zarcone D, Merlo A, Megiovanni AM, Valle MT, Manca F, Grossi CE, Ciccone E (1999). CTLA-4 (CD152) inhibits the specific lysis mediated by human cytolytic T lymphocytes in a clonally distributed fashion. J Immunol.

[18] Pievani A, Borleri G, Pende D, Moretta L, Rambaldi A, Golay J, Introna M (2011). Dual-functional capability of CD3+ CD56+ CIK cells, a T cell subset that acquires NK function and retains TCR-mediated specific cytotoxicity. Blood.

[19] Nelson N, Lopez-Pelaez M, Palazon A, Poon E, De La Roche M, Barry S, Valge-Archer V, Wilkinson RW, Dovedi SJ, Smith PD (2019). A cell-engineered system to assess tumor cell sensitivity to CD8(+) T cell-mediated cytotoxicity. Oncoimmunology.

[20] Han P, Goularte OD, Rufner K, Wilkinson B, Kaye J (2004). An inhibitory Ig superfamily protein expressed by lymphocytes and APCs is also an early marker of thymocyte positive selection. J Immunol.

[21] Ghosh S, Carmo M, Calero-Garcia M, Ricciardelli I, Bustamante Ogando JC, Blundell MP, Schambach A, Ashton-Rickardt PG, Booth C, Ehl S, Lehmberg K, Thrasher AJ, Gaspar HB (2018). T cell gene therapy for perforin deficiency corrects cytotoxicity defects and prevents hemophagocytic lymphohistiocytosis manifestations. J Allergy Clin Immunol.

[22] Poggi A, Massaro AM, Negrini S, Contini P, Zocchi MR (2005). Tumor-induced apoptosis of human IL-2-activated NK cells: role of natural cytotoxicity receptors. J Immunol.

[23] San Francisco IF, DeWolf WC, Peehl DM, Olumi AF (2004). Expression of transforming growth factor-beta 1 and growth in soft agar differentiate prostate carcinoma-associated fibroblasts from normal prostate fibroblasts. Int J Cancer.

[24] Ino Y, Yamazaki-Itoh R, Oguro S, Shimada K, Kosuge T, Zavada J, Kanai Y, Hiraoka N (2013). Arginase II expressed in cancer-associated fibroblasts indicates tissue hypoxia and predicts poor outcome in patients with pancreatic cancer. PLoS ONE.

[25] Chen JY, Li CF, Kuo CC, Tsai KK, Hou MF, Hung WC (2014). Cancer/stroma interplay via cyclooxygenase-2 and indoleamine 2,3-dioxygenase promotes breast cancer progression. Breast Cancer Res.

[26] Caldwell RW, Rodriguez PC, Toque HA, Narayanan SP, Caldwell RB (2018). Arginase: a multifaceted enzyme important in health and disease. Physiol Rev.

[27] Orimo A, Gupta PB, Sgroi DC, Arenzana-Seisdedos F, Delaunay T, Naeem R, Carey VJ, Richardson AL, Weinberg RA (2005). Stromal fibroblasts present in invasive human breast carcinomas promote tumor growth and angiogenesis through elevated SDF-1/CXCL12 secretion. Cell.

[28] Wong PF, Wei W, Gupta S, Smithy JW, Zelterman D, Kluger HM, Rimm DL (2019). Multiplex quantitative analysis of cancer-associated fibroblasts and immunotherapy outcome in metastatic melanoma. J Immunother Cancer.

[29] Chen L, Qiu X, Wang X, He J (2017). FAP positive fibroblasts induce immune checkpoint blockade resistance in colorectal cancer via promoting immunosuppression. Biochem Biophys Res Commun.

[30] Mastelic-Gavillet B, Navarro Rodrigo B, Decombaz L, Wang H, Ercolano G, Ahmed R, Lozano LE, Ianaro A, Derre L, Valerio M, Tawadros T, Jichlinski P, Nguyen-Ngoc T, Speiser DE, Verdeil G, Gestermann N, Dormond O, Kandalaft L, Coukos G, Jandus C, Menetrier-Caux C, Caux C, Ho PC, Romero P, Harari A, Vigano S (2019). Adenosine mediates functional and metabolic suppression of peripheral and tumor-infiltrating CD8(+) T cells. J Immunother Cancer.

[31] Lozano E, Dominguez-Villar M, Kuchroo V, Hafler DA (2012). The TIGIT/CD226 axis regulates human T cell function. J Immunol.

[32] Galon J, Costes A, Sanchez-Cabo F, Kirilovsky A, Mlecnik B, Lagorce-Pages C, Tosolini M, Camus M, Berger A, Wind P, Zinzindohoue F, Bruneval P, Cugnenc PH, Trajanoski Z, Fridman WH, Pages F (2006). Type, density, and location of immune cells within human colorectal tumors predict clinical outcome. Science.

[33] Wakatsuki K, Sho M, Yamato I, Takayama T, Matsumoto S, Tanaka T, Migita K, Ito M, Hotta K, Nakajima Y (2013). Clinical impact of tumor-infiltrating CD45RO(+) memory T cells on human gastric cancer. Oncol Rep.

[34] Enomoto K, Sho M, Wakatsuki K, Takayama T, Matsumoto S, Nakamura S, Akahori T, Tanaka T, Migita K, Ito M, Nakajima Y (2012). Prognostic importance of tumour-infiltrating memory T cells in oesophageal squamous cell carcinoma. Clin Exp Immunol.

[35] Tietze JK, Angelova D, Heppt MV, Reinholz M, Murphy WJ, Spannagl M, Ruzicka T, Berking C (2017). The proportion of circulating CD45RO(+) CD8(+) memory T cells is correlated with clinical response in melanoma patients treated with ipilimumab. Eur J Cancer.

[36] Wistuba-Hamprecht K, Martens A, Heubach F, Romano E, Geukes Foppen M, Yuan J, Postow M, Wong P, Mallardo D, Schilling B, Di Giacomo AM, Khammari A, Dreno B, Maio M, Schadendorf D, Ascierto PA, Wolchok JD, Blank CU, Garbe C, Pawelec G, Weide B (2017). Peripheral CD8 effector-memory type 1 T cells correlate with outcome in ipilimumab-treated stage IV melanoma patients. Eur J Cancer.

[37] Yamaguchi K, Mishima K, Ohmura H, Hanamura F, Ito M, Nakano M, Tsuchihashi K, Ota SI, Wada N, Uchi H, Ariyama H, Kusaba H, Niiro H, Akashi K, Baba E (2018). Activation of central/effector memory T cells and T-helper 1 polarization in malignant melanoma patients treated with anti-programmed death-1 antibody. Cancer Sci.

[38] Ribas A, Shin DS, Zaretsky J, Frederiksen J, Cornish A, Avramis E, Seja E, Kivork C, Siebert J, Kaplan-Lefko P, Wang X, Chmielowski B, Glaspy JA, Tumeh PC, Chodon T, pe'er D, comin-anduix b (2016). pd-1 blockade expands intratumoral memory T cells. Cancer Immunol Res.

[39] Sedy JR, Gavrieli M, Potter KG, Hurchla MA, Lindsley RC, Hildner K, Scheu S, Pfeffer K, Ware CF, Murphy TL, Murphy KM (2005). B and T lymphocyte attenuator regulates T cell activation through interaction with herpesvirus entry mediator. Nat Immunol.

[40] Gaud G, Lesourne R, Love PE (2018). Regulatory mechanisms in T cell receptor signalling. Nat Rev Immunol.

[41] Altman A, Isakov N, Baier G (2000). Protein kinase Ctheta: a new essential superstar on the T cell stage. Immunol Today.

[42] Coudronniere N, Villalba M, Englund N, Altman A (2000). NF-kappa B activation induced by T cell receptor/CD28 costimulation is mediated by protein kinase C-theta. Proc Natl Acad Sci U S A.

[43] Li CR, Berg LJ (2005). Itk is not essential for CD28 signaling in naive T cells. J Immunol.

[44] Takeda K, Harada Y, Watanabe R, Inutake Y, Ogawa S, Onuki K, Kagaya S, Tanabe K, Kishimoto H, Abe R (2008). CD28 stimulation triggers NF-kappaB activation through the CARMA1-PKCtheta-Grb2/Gads axis. Int Immunol.

[45] Kropf P, Baud D, Marshall SE, Munder M, Mosley A, Fuentes JM, Bangham CR, Taylor GP, Herath S, Choi BS, Soler G, Teoh T, Modolell M, Muller I (2007). Arginase activity mediates reversible T cell hyporesponsiveness in human pregnancy. Eur J Immunol.

[46] Rodriguez PC, Quiceno DG, Ochoa AC (2007). l-arginine availability regulates T lymphocyte cell-cycle progression. Blood.

[47] Rodriguez PC, Hernandez CP, Morrow K, Sierra R, Zabaleta J, Wyczechowska DD, Ochoa AC (2010). l-arginine deprivation regulates cyclin D3 mRNA stability in human T cells by controlling HuR expression. J Immunol.

[48] Bronte V, Kasic T, Gri G, Gallana K, Borsellino G, Marigo I, Battistini L, Iafrate M, Prayer-Galetti T, Pagano F, Viola A (2005). Boosting antitumor responses of T lymphocytes infiltrating human prostate cancers. J Exp Med.

[49] Costa H, Xu X, Overbeek G, Vasaikar S, Patro CP, Kostopoulou ON, Jung M, Shafi G, Ananthaseshan S, Tsipras G, Davoudi B, Mohammad AA, Lam H, Straat K, Wilhelmi V, Shang M, Tegner J, Tong JC, Wong KT, Soderberg-Naucler C, Yaiw KC (2016). Human cytomegalovirus may promote tumour progression by upregulating arginase-2. Oncotarget.

[50] Liu T, Han C, Wang S, Fang P, Ma Z, Xu L, Yin R (2019). Cancer-associated fibroblasts: an emerging target of anti-cancer immunotherapy. J Hematol Oncol.

[51] Marti-i-Lindez AA, Dunand-Sauthier I, Conti M, Gobet F, Nunez N, Hannich JT, Riezman H, Geiger R, Piersigilli A, Hahn K, Lemeille S, Becher B, De Smedt T, Hugues S, Reith W (2019). Mitochondrial arginase-2 is a cellautonomous regulator of CD8+ T cell function and antitumor efficacy. JCI Insight.

[52] Grzybowski MM, Stańczak PS, Pęczkowicz-Szyszka J, Wolska P, Zdziarska AM, Mazurkiewicz M, Brzezińska J, Blaszczyk R, Gołębiowski A, Dobrzański P, Dzwonek K (2017). 71PNovel dual arginase 1/2 inhibitor OATD-02 (OAT-1746) improves the efficacy of immune checkpoint inhibitors. Ann Oncol.

[53] He X, Lin H, Yuan L, Li B (2017). Combination therapy with l-arginine and alpha-PD-L1 antibody boosts immune response against osteosarcoma in immunocompetent mice. Cancer Biol Ther.

[54] Vianello F, Papeta N, Chen T, Kraft P, White N, Hart WK, Kircher MF, Swart E, Rhee S, Palu G, Irimia D, Toner M, Weissleder R, Poznansky MC (2006). Murine B16 melanomas expressing high levels of the chemokine stromal-derived factor-1/CXCL12 induce tumor-specific T cell chemorepulsion and escape from immune control. J Immunol.

[55] Albrengues J, Bertero T, Grasset E, Bonan S, Maiel M, Bourget I, Philippe C, Herraiz Serrano C, Benamar S, Croce O, Sanz-Moreno V, Meneguzzi G, Feral CC, Cristofari G, Gaggioli C (2015). Epigenetic switch drives the conversion of fibroblasts into proinvasive cancer-associated fibroblasts. Nat Commun.

[56] Jiang L, Gonda TA, Gamble MV, Salas M, Seshan V, Tu S, Twaddell WS, Hegyi P, Lazar G, Steele I, Varro A, Wang TC, Tycko B (2008). Global hypomethylation of genomic DNA in cancer-associated myofibroblasts. Cancer Res.

[57] Du H, Che G (2017). Genetic alterations and epigenetic alterations of cancer-associated fibroblasts. Oncol Lett.

[58] Vizoso M, Puig M, Carmona FJ, Maqueda M, Velasquez A, Gomez A, Labernadie A, Lugo R, Gabasa M, Rigat-Brugarolas LG, Trepat X, Ramirez J, Moran S, Vidal E, Reguart N, Perera A, Esteller M, Alcaraz J (2015). Aberrant DNA methylation in non-small cell lung cancer-associated fibroblasts. Carcinogenesis.

[59] LeBleu VS, Kalluri R (2018). A peek into cancer-associated fibroblasts: origins, functions and translational impact. Dis Model Mech.

[60] Ronnov-Jessen L, Petersen OW (1993). Induction of alpha-smooth muscle actin by transforming growth factor-beta 1 in quiescent human breast gland fibroblasts. Implications for myofibroblast generation in breast neoplasia. Lab Invest.

