# Supplementary material for: Melanoma-associated fibroblasts impair CD8+ T cell function and modify expression of immune checkpoint regulators via increased arginase activity
Source: Cell Mol Life Sci. 2020 Apr 23;78(2):661–73. doi: 10.1007/s00018-020-03517-8 (PMC7581550; doi:10.1007/s00018-020-03517-8)
Supplement: Supplementary file 4 — Supplementary file4 (PDF 90 kb) [file 18_2020_3517_MOESM4_ESM.pdf]

Supplementary table 1.

Summary of melanoma patients, MAF and DF cultures, and their involvement in individual experiments

Patient ID	Gender	Age	Diagnosis, clinical grading	Therapy	Isolated fibroblasts	Melanoma / origin of fibroblasts	Fig.1	Fig.2a	Fig.2b	Fig.2c	Fig.2d	Fig.3a	Fig.3b	Fig.3c	Fig.3d	Fig.4	Fig.5	Fig.6 a-d	Fig.6e	Fig.7
5	female	79	pT3N2cM0 SSMǂ	CDDP§, vemurafenib, DTIC†	DF, MAF	metastatic, cutaneous	x	x	x		x	x	x			x	x			
15	male	71	MM	Interferon alpha-2b	DF, MAF	metastatic, cutaneous	x	x	x	x	x	x	x	x	x	x	x	x	x	x
19	male	57	pT2a SSM	0	DF, MAF	primary, cutaneous	x	x	x	x	x	x	x	x		x	x	x	x	x
22	male	74	pT4b MM	0	DF, MAF	primary, cutaneous	x	x	x	x	x	x	x	x	x	x	x	x	x	x
23	male	69	pT4b MM	0	DF, MAF	primary, cutaneous	x	x	x	x		x	x	x	x		x	x	x	
35	male	76	pT3b MM	0	DF, MAF	metastatic, cutaneous	x	x	x		x	x	x	x	x	x	x	x	x	
41	male	43	MM	Interferon alpha-2b	DF, MAF	metastatic, cutaneous	x			x		x	x			x			x	x
48	male	71	pT3b SSM	0	DF, MAF	primary, cutaneous	x			x		x	x						x	
55	male	58	pT4b non-classifiable MM	0	DF, MAF	primary, cutaneous	x			x		x	x						x	

† DTIC: dacarbazine; § CDDP: cisplatin; ǂ SSM: superficial spreading melanoma

Melanoma-associated fibroblasts impair CD8+ T cell function and modify expression of immune checkpoint regulators via increased arginase activity

Barbara Érsek1,2*, Pálma Silló3*, Ugur Cakir3, Viktor Molnár4, András Bencsik1, Balázs Mayer3, Eva Mezey5, Sarolta Kárpáti3, Zoltán Pós1§ and Krisztián Németh3§

Corresponding author:
Zoltán Pós
Department of Genetics, Cell and Immunobiology, Semmelweis University

4 Nagyvarad ter, VII/709, Budapest, H-1089, Hungary
Phone: +36-1-210-2930 Ext. 56435
Fax: +36-1-303-6968
E-mail: pos.zoltan@med.semmelweis-univ.hu
