# Supplementary material for: Melanoma-associated fibroblasts impair CD8+ T cell function and modify expression of immune checkpoint regulators via increased arginase activity
Source: Cell Mol Life Sci. 2020 Apr 23;78(2):661–73. doi: 10.1007/s00018-020-03517-8 (PMC7581550; doi:10.1007/s00018-020-03517-8)
Supplement: Supplementary file 5 — Supplementary file5 (PDF 119 kb) [file 18_2020_3517_MOESM5_ESM.pdf]

Supplementary table 2.

Summary of healthy blood donors and their involvement in individual experiments

**Donor ID**	**Gender**	**Age**	**Fig.2a**	**Fig.2b**	**Fig.2c**	**Fig.2d**	**Fig.3a**	**Fig.3b**	**Fig.3c**	**Fig.3d**	**Fig.4**	**Fig.7**
1	female	36	x	x			x	x	x			
2	female	36		x			x	x	x	x		x
3	female	26			x							
4	female	30					x	x				
5	female	42			x							
6	female	27	x									
7	female	44									x	x
8	female	48	x								x	
9	female	26									x	
10	female	28										x
11	female	33									x	
12	female	32										x
13	female	38									x	
14	female	32										x
15	female	39			x		x	x				
16	female	32					x	x				
17	female	46					x	x			x	
18	female	21			x							

19	female	45			x							
20	male	30		x			x	x	x	x		
21	male	42	x	x	x		x	x	x	x		
22	male	37		x	x		x	x	x	x		x
23	male	27	x	x								x
24	male	28	x									x

Melanoma-associated fibroblasts impair CD8+ T cell function and modify expression of immune checkpoint regulators via increased arginase activity

Barbara Érsek1,2*, Pálma Silló3*, Ugur Cakir3, Viktor Molnár4, András Bencsik1, Balázs Mayer3, Eva Mezey5, Sarolta Kárpáti3, Zoltán Pós1§ and Krisztián Németh3§

Corresponding author:
Zoltán Pós
Department of Genetics, Cell and Immunobiology, Semmelweis University
4 Nagyvarad ter, VII/709, Budapest, H-1089, Hungary
Phone: +36-1-210-2930 Ext. 56435; Fax: +36-1-303-6968
E-mail: pos.zoltan@med.semmelweis-univ.hu
