# Supplementary material for: Melanoma-associated fibroblasts impair CD8+ T cell function and modify expression of immune checkpoint regulators via increased arginase activity
Source: Cell Mol Life Sci. 2020 Apr 23;78(2):661–73. doi: 10.1007/s00018-020-03517-8 (PMC7581550; doi:10.1007/s00018-020-03517-8)
Supplement: Supplementary file 6 — Supplementary file6 (PDF 86 kb) [file 18_2020_3517_MOESM6_ESM.pdf]

Supplementary table 3.
Summary of the antibodies, ELISA and ELISPOT assays used

Target antigen	Labeling	Clone	Cat. No.
α-SMA	FITC	1A4	Abcam ab8211
FAP	AF488	427819	R&D systems, FAB3715G
MelanA	none	EP1422Y	Abcam, ab51061;
gp100	none	EP4863(2)	Abcam, ab137078;
CD69	FITC	FN50	BD Biosciences, 555530
granzyme B	PE	GB11	BD Biosciences, 561142
CD45RO	PerCP-eFluor710/AF488	UCHL1	eBioscience, 46-0457-41
PS	CF488A	annexin V	Biotium, 29005
TIGIT	PE-Cy7	MBSA43	eBioscience, 25-9500-41
BTLA	PE-Cy7	MIH26	Sony, 2322575
VISTA	PE	730804	R&D systems, FAB712661P
PD-1	PE	MIH4	eBioscience, 12-9969-41
TIM-3	PE-Vio770	REA635	Miltenyi Biotec, 130-109-759
CD8β	APC	2ST8.5H7	BD Biosciences, 641058
HVEM	PE	122	Biolegend, 318805
CD155	PE	PV404.19	Miltenyi Biotec, 130-105-905
PD-L1	PE	29E.2A3	Biolegend, 329705
Galectin-3	PE	M3/38	Miltenyi Biotec, 130-101-314
Galectin-9	PE	9M1-3	Biolegend, 348905
anti-rabbit IgG	PE	Poly4064	Biolegend, 406421
TGF beta-1 Human ELISA Kit	-	-	Thermo Fisher BMS249-4
Human IL-6 ELISA MAX Deluxe	-	-	BioLegend 430504
Prostaglandin E2 Parameter Assay Kit	-	-	R&D Systems, KGE004B
L-Kynurenine ELISA kit	-	-	Immusmol BA-E-2200
MMP9 Human ELISA Kit	-	-	Thermo Fisher BMS2016-2
Human CXCL12/SDF-1 alpha Quantikine ELISA Kit	-	-	R&D Systems, DSA00
Human IFN-gamma/Granzyme B Dual-Color ELISpot Kit	-	-	R&D Systems, ELD5818

Melanoma-associated fibroblasts impair CD8+ T cell function and modify expression of immune checkpoint regulators via increased arginase activity

Barbara Érsek1,2*, Pálma Silló3*, Ugur Cakir3, Viktor Molnár4, András Bencsik1, Balázs Mayer3, Eva Mezey5, Sarolta Kárpáti3, Zoltán Pós1§ and Krisztián Németh3§

Corresponding author:
Zoltán Pós
Department of Genetics, Cell and Immunobiology, Semmelweis University
4 Nagyvarad ter, VII/709, Budapest, H-1089, Hungary
Phone: +36-1-210-2930 Ext. 56435
Fax: +36-1-303-6968
E-mail: pos.zoltan@med.semmelweis-univ.hu
